# The Urokinase/Urokinase Receptor System in Mast Cells: Effects of its Functional Interaction with fMLF Receptors

**Published:** 2016-11-01

**Authors:** Francesca Wanda Rossi, Nella Prevete, Felice Rivellese, Filomena Napolitano, Nunzia Montuori, Loredana Postiglione, Carmine Selleri, Amato de Paulis

**Affiliations:** 1 Department of Translational Medical Sciences, Federico II University of Naples, Naples, Italy; 2 Centre for Experimental Medicine and Rheumatology, William Harvey Research Institute, Barts and The London, School of Medicine and Dentistry, Queen Mary University of London, London, UK; 3 Department of Medicine and Surgery, University of Salerno, Baronissi, Italy

**Keywords:** Mast Cells, FPRs, uPA/uPAR, VEGF-A

## Abstract

Mast cell and basophils express the high affinity receptor for IgE (FcɛRI) and are primary effector cells of allergic disorders. The urokinase (uPA)-mediated plasminogen activation system is involved in physiological and pathological events based on cell migration and tissue remodelling, such as inflammation, wound healing, angiogenesis and metastasis. uPA is a serine protease that binds uPAR, a high affinity glycosyl-phosphatidyl-inositol (GPI)-anchored receptor. uPAR focuses uPA activity at the cell surface and activates intracellular signaling through lateral interactions with integrins, receptor tyrosine kinases and the G-protein-coupled family of fMLF chemotaxis receptors (FPRs).

We investigated the expression of the uPA-uPAR system and its functional interaction with FPRs in human mast cells (MCs). Differently from basophils, MCs produced uPA that was able to induce their chemotaxis. Indeed, MCs also expressed uPAR, both in the intact and in a cleaved form (DII-DIII-uPAR) that can expose, at the N-terminus, the SRSRY sequence, able to interact with FPRs and to mediate cell chemotaxis. MCs also expressed mRNAs for FPRs that were functionally active; indeed, uPA and a soluble peptide (uPAR_84–95_), containing the SRSRY chemotactic sequence of uPAR and able to interact with FPRs, were able to induce MCs chemotaxis.

Thus, uPA is a potent chemoattractant for MCs acting through the exposure of the chemotactic epitope of uPAR, that is an endogenous ligand for FPRs. The same mechanism could be involved in VEGF-A secretion by human MCs, also induced by uPA and uPAR_84–95_ stimulation.

## I. INTRODUCTION

Mast cells (MCs) are haematopoietic cells widely distributed in vascularized tissues, at the interface with the external environment. Unlike other immune cells, MCs normally circulate through the vascular system as immature progenitors and undergo the terminal stages of their differentiation and/or maturation locally, after migration into vascularized tissues or serosal cavities, in a process regulated by multiple local factors [1]. The main factors that influence MCs number and phenotype include c-Kit ligand stem-cell factor (SCF) [2] and the chief survival and/or developmental factors for the MCs, IL-3 and T helper type 2 (Th2)-associated cytokines such as IL-4 and IL-9, but the complete list comprises a wide panel of other growth factors, cytokines and chemokines [1].

MCs abound especially near surfaces exposed to the environment, including the gastrointestinal and airways tract and skin, where pathogens, allergens and other environmental agents are frequently encountered [3–5]. Due to their specific anatomical location, MCs have numerous functions; in particular they are responsible for the first line of defence against external pathogens and other environmental insults [6]. MCs are well known for their versatile role in allergic responses through the binding of specific antigens to the FcɛRI-IgE complex [1]. However, in recent years, it has been demonstrated that MCs contribute to a variety of non-allergic immunoregulatory reactions. MCs infiltrate the sites of chronic inflammation [7]; increased numbers of MCs have been found in the synovial tissues and fluids of patients with rheumatoid arthritis (RA), and at sites of cartilage erosion [8]. It has been reported that MC density is increased at the margins of various tumors in humans [9, 10], modulating many aspects of the tumor natural history [11–13] and correlating with angiogenesis through the synthesis and release of a wide spectrum of angiogenic factors, such as Vascular Endothelial Growth Factor-A (VEGF-A) and Vascular Endothelial Growth Factor-B (VEGF-B), [14,15] and tumor invasion by releasing cytokines and proteases [12].

The urokinase-type plasminogen activator receptor (uPAR, CD87) is a GPI-anchored protein that functions as the receptor for urokinase (uPA) [16]. uPAR, expressed by a wide variety of cells, including monocytes, macrophages, neutrophils and basophils [17,18], is formed by three homologous domains (DI, DII, DIII) [19]. The uPAR can be cleaved within the DI/DII linker region by several proteolytic enzymes, including uPA itself [20,21]. The cleavage causes the release of DI from the molecule. Therefore, uPAR can exist on the cell surface in either a three-domain form (DI-DII-DIII-uPAR), which is capable of binding uPA, or a two-domain form (DII-DIII-uPAR), which does not bind uPA [20].

uPAR has important roles in both physiological and pathological processes; in addition to its regulatory role in fibrinolysis and inflammation, it has been implicated in tumor invasion, metastasis, fibrosis, and in the development of protective immunity in infections. In particular, uPAR is strongly up-regulated in several cancers where represents a negative prognostic factor [22]. uPAR traditional role was considered the focusing of proteolytic uPA activity on the cell membrane, however uPAR also binds vitronectin (VN), a component abundant in tumor-associated ECM [23], and interacts with various integrins regulating their activity. In addition uPAR mediates uPA-dependent cell migration and is required for chemotaxis induced by fMet-Leu-Phe (fMLF), a potent leukocyte chemoattractant. Through a specific site corresponding to amino acids 88–92 (SRSRY), located in the region linking uPAR domain 1 (DI) to uPAR domain 2 (DII), the cell-surface uPAR functionally interacts with the *N*-formyl peptide receptors (FPRs) [24].

FPRs are a family of pattern recognition receptors. It is now well known that, by interacting with several structurally diverse pro- and anti-inflammatory ligands, FPRs seem to possess important regulatory effects in multiple pathological conditions, including inflammation and cancer. FPRs are expressed in abundance on cells of the host defense system; in addition, all FPRs expressed on epithelia seem to be required for wound repair and restitution of barrier integrity, by facilitating epithelial cell migration, proliferation, and neo-angiogenesis [25]. Three variants of FPRs have been identified in humans: the high and low affinity receptors, FPR1 and FPR2, and the FPR3, which does not bind fMLF [26].

Recently, new insight on the diversity of MC products, signalling mechanisms, and interactions with other cell types has led to many attractive hypotheses about the diverse potential effector and immunoregulatory roles of MCs in physiological and pathological conditions. In particular, several authors focused their attention on the role of MCs in tumor growth, starting from the observation that mast cell deficient mice show a reduced cancer infiltration [27]. Based on such evidences, in this study we investigated whether MCs, by expressing and modulating the FPRs and the uPA/uPAR system, could represent a novel target in several inflammatory and neoplastic diseases.

## II. METHODOLOGY

### Peptides and chemicals

The following were purchased: di-isopropyl fluorophosphate (DFP; Fluka, Buchs, Switzerland); fMLF was from Calbiochem (La Jolla, CA); the peptide uPAR_84–95_ was synthesized by PRIMM (Milan, Italy), the 59-(N-ethylcarboxamido) adenosine (NECA) was from Sigma-Aldrich (St. Louis, MO, USA), the phorbol myristate acetate (PMA) was obtained from LC Services (Woburn, MA), human uPA and the uPA N-terminal fragment (ATF) were from Sekisui Diagnostics (Lexington, MA, USA); PE-labeled anti-IgE Abs (Caltag Laboratories, Burlingame, CA); FITC-labeled goat anti-rabbit IgG (Abcam, Cambridge, U.K.). For chemotaxis assay 8-mm-pore polycarbonate membranes (Nucleopore, Pleasanton, CA), TRIzol solution was from Invitrogen FischerScientific (Illkirch, France), and DNA ladder and Moloney leukemia virus reverse transcriptase were from Promega (Madison, WI). Protein concentration was estimated with a modified Bradford assay (Bio-Rad Laboratories). ECL Plus was from GE Healthcare (Buckinghamshire, UK). The mixture of protease and phosphatase inhibitors was from Calbiochem. Rabbit polyclonal anti-uPAR and monoclonal anti-uPA antibodies were from Sekisui Diagnostics, rabbit anti-actin was from Sigma-Aldrich (St. Louis, MO). Secondary anti-mouse and anti-rabbit Abs coupled to HRP were from Bio-Rad (Munchen, Germany). RANTES was from PeproTech EC LTD (London, UK) and SCF was from recombinant human stem cell factor (SCF) from Amgen (Thousand Oaks, CA).

### Cell culture

Human mast cell line HMC-1 was kindly donated by Dr. J.H. Butterfield (Mayo Clinic, Rochester, MN); cells were maintained in suspension culture at a density of 3–9 × 10^5^ cells/ml of IMDM supplemented with 10% FCS, 2 mM L-glutamine, 1.2 mM monothioglicerol. The THP-1 monocyte-like cell line was grown in RPMI 1640 medium supplemented with 10% heat-inactivated FCS [17,18].

### Isolation and purification of human lung mast cells (HLMC)

Lung tissue was obtained from patients undergoing thoracotomy and lung resection, after obtaining their informed consent according to the guidelines of the institutional review board. Macroscopically normal parenchyma was dissected free from pleura, bronchi, and blood vessels and minced into a single-cell suspension as previously described [28]. Yields ranged between 3×10^6^ and 18×10^6^ mast cells, and purity was between 1 and 8%. Lung mast cells were purified by countercurrent elutriation (J2/21; Beckman) and then by discontinuous Percoll density gradient as previously described. Mast cells were further purified to near homogeneity by positive selection: incubation with anti-FcɛRI (IgG1) was followed by the exposure to magnetic beads coated with MACS goat anti-mouse IgG. Labeled cells were enriched by positive selection columns (MACS system; Miltenyi Biotec). The final preparations contained >95% viable cells, as assessed by the trypan blue exclusion method, and purity was >98% mast cells.

### RNA purification and analysis

Total cellular RNA was isolated by lysing cells in TRIzol solution, according to the supplier’s protocol [29]. RNA was precipitated and quantitated by spectroscopy. Five micrograms of total RNA was reversely transcribed with random hexamer primers and 200 U murine Moloney leukemia virus reverse transcriptase. One microliter of reverse-transcribed DNA was then amplified for FPR1, FPR2, FPR3, uPA, uPAR and GAPDH using specific primers. The primers for FPR1 were 5′-ATGGAGACAAATTCCTCTCTC (sense) and 3′-CACCTCTGCAGAAGGTAAAGT (antisense); for FPR2 were 5′-CTTGTGATCTGGGTGGCTGGA (sense) and 3′CATTGCCTGTAACTCAGTCTC (antisense); and for FPR3 were 5′AGTTGCTCCACAGGAATCCA (sense) and 3′-GCCAATATTGAAGTGGAGGATCAGA (antisense), for uPA were 5′-AAAATGCTATGTGCTGCTGACC (sense) and 3′CCCTGCCCTGAAGTCGTTAGTG (antisense) [24], for uPAR were 5′CTGCGGTGCATGCAGTGTAAG (sense) and 3′GGTCCAGAGGAGAGTGCCTCC (antisense) [18], for VEGF-A were 5′TCTTCAAGCCATCCTGTGTG (sense) and 3′GCCTCGGCTTGTCACATC (antisense) [30].

The primers for GAPDH were 5′GCCAAAGGGTCATCATCTC (sense) and 3′-GTAGAGGCAGGGATGATGTTC (antisense). PCR products, together with a DNA ladder as a size standard, were separated on a 1% agarose gel, stained with ethidium bromide, and quantified with the image analysis system ChemiDoc XRSn (Bio-Rad Laboratories).

### Western blot

Immunoblotting experiments were performed according to standard procedures [31]. Briefly, cells were harvested in lysis buffer (50 mM HEPES, 150 mM NaCl, 10% glycerol, 1% Triton X-100, 1 mM EGTA, 1.5 mM MgCl2, 10 mM NaF, 10 mM sodium pyrophosphate, and 1 mM Na3VO4) supplemented with a mixture of proteases and phosphatases inhibitors. The protein content was measured by a colorimetric assay. Fifty micrograms of protein was electrophoresed on a 10% SDS-PAGE under non reducing conditions and transferred onto a polyvinylidene fluoride membrane.

The membrane was blocked with 5% nonfat dry milk and probed with specific Abs: mouse anti-uPA (1μg/ml), rabbit anti uPAR (1μg/ml), and rabbit anti-β-actin (0.5 μg/ml). Finally, washed filters were incubated with HRP-conjugated anti-rabbit or antimouse Abs. The immunoreactive bands were detected by a chemiluminescence kit and quantified by densitometry (ChemiDoc XRS, BioRad).

### Flow cytometric analysis of surface molecules

Flow cytometric analysis of cell surface molecules was performed as previously described [18]. Briefly, after saturation of non specific binding sites with total rabbit IgG, cells were incubated for 20 min at +4°C with specific or isotype control antibodies. For indirect staining this step was followed by a second incubation for 20 min at +4°C with an appropriate anti-isotype-conjugated antibody. Finally, cells were washed and analyzed with a FACSCalibur Cytofluorometer using Cell Quest software (Becton & Dickinson, San Fernando, CA). A total of 10^4^ events for each sample were acquired in all cytofluorimetric analyses.

### Chemotaxis assay

Human lung mast cell (HLMC) chemotaxis was performed using a modified Boyden chamber technique as previously described [18]. Briefly, 25 μl of PACGM buffer or various concentrations of the chemoattractants in the same buffer were placed in triplicate in the lower compartment of a 48-well microchemotaxis chamber (Neuroprobe, Cabin John, MD). The lower compartments were covered with polycarbonate membranes with a two-filter sandwich constituted by 5-μm (lower) and 8-μm (upper) pore size polycarbonate membranes (Nucleopore, Pleasanton, CA). Fifty microliters of the cell suspensions (5×10^4^/well) resuspended in PACGM was pipetted into the upper compartments. The chemotactic chamber was then incubated for 3 h at 37°C in a humidified incubator with 5% CO_2_ (automatic CO_2_ incubator, model 160 IR, ICN/Flow Laboratories). At the end of the incubation the upper polycarbonate filter was discarded, while the lower nitrate cellulose filter was fixed in methanol, stained with Alcian Blue, and then mounted on a microscope slide with Cytoseal (Stephen Scientific, Springfield, NJ).

HLMC chemotaxis was quantitated microscopically by counting the number of cells attached to the surface of the 5-μm cellulose nitrate filter. In each experiment 10 fields/triplicate filter were measured at × 40 magnification. The results were compared with buffer controls.

Check board analysis was performed to discriminate between chemotaxis and nondirected migration (chemokines) of HLMC. In these experiments mast cells were placed in the upper chambers, and various concentrations of stimuli or buffer were added into the upper or lower wells or both. Spontaneous migration (chemokinesis) was determined in the absence of chemoattractant or when stimuli were added to both lower and upper chambers.

The mast cell migratory responses to the stimuli were largely due to chemotaxis and not to chemokinesis. Indeed, a check board analysis, in which chemoattractants above and below the filters were varied, resulted in significant migration only when there was a gradient of the factor below the filters (data not shown).

### ELISA for VEGF-A

VEGF-A content in cell lysates and in culture supernatants of human mast cells was measured in duplicate determinations with a commercially available ELISA (R&D Systems) [32].

### Inactivation of uPA

The uPA was inactivated by incubation with 10 mM DFP for 2 h at 4°C [18].

## III. RESULTS

### Expression of uPAR and FPRs in the human mast cell line HMC-1

In several conditions, such as in the fibroblast-to-myo fibroblast transition involved in the pathogenesis of inflammatory and neoplastic disorders, the uPA/uPAR system acts through the structural and functional interaction with FPRs [24].

**Figure 1:**
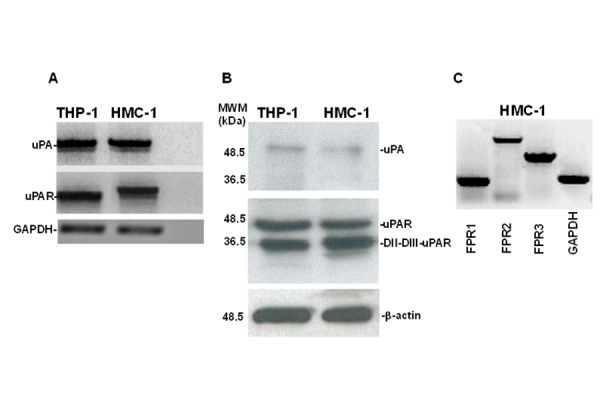
uPA, uPAR and FPRs expression in HMC-1 cells. **A**: mRNA expression of uPA and uPAR in HMC-1 cells. Total RNA of THP-1 monocyte-like cells as a positive control (lane 1) and HMC-1 cells (lane 2) were prepared, reverse transcribed, and amplified by 40 PCR cycles in the presence of uPA and uPAR-specific primers and GAPDH primers, as a loading control. PCR products were analyzed by electrophoresis in 1% agarose gel containing ethidium bromide, followed by photography under UV illumination. **B**: Western blot analysis of uPA and uPAR in HMC-1 cells. THP-1 monocyte-like cells as a positive control (lane 1) and HMC-1 cells (lane 2) were lysed in Triton X-100 and 50 μg of total protein were analyzed by 9% SDS-PAGE and Western blot with an anti-uPA and anti-uPAR polyclonal antibody. **C**: mRNA expression of FPRs in HMC-1 cells. Total RNA was prepared, reverse transcribed and amplified by 40 PCR cycles in the presence of FPR1- (lane 1), FPR2- (lane 2), and FPR3- (lane 3) specific primers and GAPDH (lane 4) primers, as loading control. PCR products were analyzed by electrophoresis in 1% agarose gel containing ethidium bromide, followed by photography under UV illumination.

To investigate the existence of a functional interaction between the uPA-uPAR system and FPRs in MCs, we first evaluated the expression at mRNA and protein level of uPA and uPAR in HMC-1 cells. uPA and uPAR mRNAs were detected by RT-PCR analysis of RNAs from HMC-1 cells and THP-1 monocyte-like cells, used as a positive control (Fig. 1A).

Western blot analysis of HMC-1 cell lysates with a monoclonal anti-uPA antibody confirmed the production of uPA. The same analysis with a polyclonal anti-uPAR antibody demonstrated that HMC-1 cells expressed uPAR in the intact (DI-DII-DIII-uPAR) and cleaved (DII-DIII-uPAR) forms (Fig. 1B). This result was previously described in other cell types, including monocyte-like THP-1 cells [18].

DII-DIII-uPAR can interact with FPRs through its SRSRY chemotactic domain, exposed at the N-terminus [21,33]. Moreover, upon binding uPA, intact uPAR can expose the same chemotactic domain, located in the DI-DII linker region, through a conformational modification [18]. Then, we examined FPRs expression in HMC-1 cells by RT-PCR analysis. Electrophoresis in agarose gel showed the presence of mRNAs for all three receptors: FPR1, FPR2 and FPR3 (Fig. 1C).

These experiments demonstrated that MCs synthesized uPA and its specific receptor uPAR as well as FPRs, most important mediators of uPA/uPAR-induced cell responses in cancer and inflammation [16,22,24,31].

### Effects of DFP-uPA and uPAR_84–95_ on VEGF-A expression and production in the HMC-1 human mast cell line

It has been reported that human mast cells can express and synthesize VEGF-A and VEGF-B, as well as Vascular Endothelial Growth Factor-C (VEGF-C) and Vascular Endothelial Growth Factor-D (VEGF-D) [14, 15].

Thus, we evaluated whether uPA, both directly and/or by inducing uPAR interaction with FPRs, could stimulate VEGF-A expression and production by HMC-1 cells.

To this aim, HMC-1 cells were stimulated for 2 hours with DFP inactivated-uPA (10^−8^ M), able to bind uPAR but devoid of enzymatic activity, and with the uPAR_84–95_ peptide (10^−9^ M), containing the SRSRY chemotactic sequence of uPAR and able to bind FPRs.

**Figure 2:**
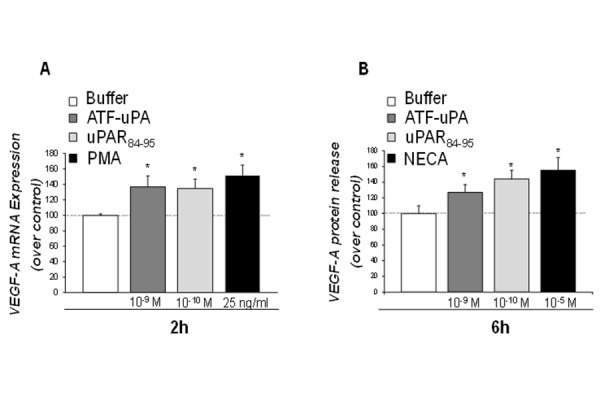
Effect of ATF-uPA and uPAR_84–95_ on VEGFA expression and release by HMC-1 cells. **A**: effect of ATF-uPA, uPAR_84–95_ on VEGF-A mRNA expression in HMC-1 cells. HMC-1 cells were cultured with cell medium alone, ATF-uPA (10^−9^ M), uPAR_84–95_ (10^−10^ M), or PMA (25 ng/ml), as a positive control, for 2 hours at 37°C in a humidified (5% CO2) incubator. Total RNA of HMC-1 cells was prepared, reverse transcribed and amplified by 40 PCR cycles in the presence of VEGF-A specific primers and GAPDH primers, as a loading control. PCR products were analyzed by electrophoresis in 1% agarose gel containing ethidium bromide, followed by photography under UV illumination. Results are expressed as percentage of VEGF-A expression increase in 10^−9^ M ATF-uPA-treated (grey column), 10^−10^ M uPAR_84–95_-treated (light grey column), or 25 ng/ml PMA-treated (black column) HMC-1 cells, relative to untreated HMC-1 cells (white column), after normalization to GAPDH. Values are the mean ± SEM of three experiments. * *p*: <0.05. **B**: effect of ATF-uPA and uPAR_84–95_ on VEGF-A synthesis by HMC-1 cells. 10^6^ cells/samples were incubated for 6 hours without (white column) or with ATF-uPA (10^−9^ M) (grey column), uPAR_84–95_ (10^−10^ M) (light grey column), or NECA (10^−5^ M) (black column) at 37°C in a humidified (5% CO2) incubator. Results are expressed as percentage of increase of VEGF-A release relative to untreated cells. HMC-1 supernatants were collected and VEGF-A was determined by ELISA assay. Values are the mean ± SEM of three experiments. * *p*: <0.05.

RT-PCR analysis of VEGF-A mRNA expression showed that HMC-1 cells synthetized mRNA for VEGF-A after 2 hours stimulation with both ligands (Fig. 2A).

To evaluate whether DFP-uPA and uPAR_84–95_ also induced VEGF-A production, we investigated the kinetics of VEGF-A release from HMC-1 cells. Both DFP-uPA and uPAR_84–95_ induced a time-dependent release of VEGF-A in HMC-1 cells (not shown) that was significant after 6 hours of stimulation (Fig 2B).

### Effect of DFP-uPA, and uPAR_84–95_ on primary human lung mast cells (HLMC) chemotaxis

We demonstrated that uPA can induce human basophil chemotaxis through uPAR functional interaction with FPRs [18].

**Figure 3:**
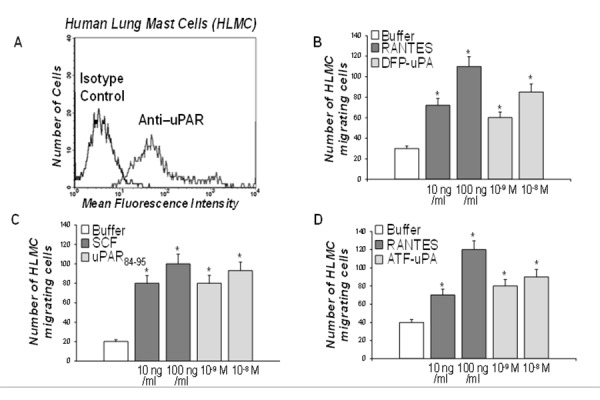
uPAR expression and function in primary HLMC cells. **A**: cytofluorometric analysis of uPAR expression on HLMC cell surface. HLMC were incubated with anti-IgE PE monoclonal antibody, anti-uPAR polyclonal antibody, isotype-matched control polyclonal antibody and FITC-conjugated goat anti-rabbit antibody. **B**: effect of DFP-uPA on HLMC cell chemotaxis. HLMC were allowed to migrate in response to cell medium alone (white column), or with the indicated concentrations of RANTES (grey column) or DFP-uPA (light grey column) for 3 h at 37°C in a humidified (5% CO2) incubator. Values are the mean ± SEM of three experiments. *p: <0.05. **C**: effect of uPAR_84–95_ on HLMC cell chemotaxis. HLMC were allowed to migrate in response to cell medium alone (white column), or with the indicated concentrations of SCF (grey column) or uPAR_84–95_ (light grey column) for 3 h at 37°C in a humidified (5% CO2) incubator. Values are the mean ± SEM of three experiments. * *p*: <0.05. **D**: effect of ATF-uPA on HLMC cell chemotaxis. HLMC were allowed to migrate in response to cell medium alone (white column), or with the indicated concentrations of RANTES (grey column) or ATF-uPA (light grey column) for 3 h at 37°C in a humidified (5% CO2) incubator. Values are the mean ± SEM of three experiments. * *p*: <0.05.

Thus, we the studied uPAR expression and the existence of a functional interaction with FPRs also in primary human MCs. To this aim, human lung mast cells (HLMC) were purified from normal subjects and analyzed by flow cytometry with a polyclonal anti-uPAR antibody or with purified control IgG, then stained with FITC-conjugated goat anti-rabbit IgG and PE-conjugated anti-IgE. The vast majority (80–94%) of HLMC showed uPAR expression on the cell surface (Fig. 3A).

In order to elucidate the potential interaction of cell-surface uPAR and FPRs, we tested the effects of DFP-inactivated uPA (10^−9^–10^−8^ M) and of different concentrations of the uPAR-derived chemotactic peptide uPAR_84–95_ (10^−9^–10^−8^ M) on purified HLMC chemotaxis. Both DFP-uPA and uPAR_84–95_ caused HLMC migration (Fig. 3B, C). Both the chemokine RANTES (10 and 100 ng/ml) and the major chemotactic factor SCF (10 and 100 ng/ml), used as a positive control, showed to be potent factors in HLMC migratory activity (Fig. 3B, C).

To determine whether migration of HLMC resulted from chemotaxis or chemokinesis, checkerboard analysis was performed and showed that both stimuli in a dose dependent manner induced HLMC migration when added to the lower wells of the chemotaxis chamber. An optimal concentration of the stimuli added with the cells to the upper wells or to both compartments did not induce directional HLMC migration. Thus, DFP-uPA and uPAR_84–95_-induced migration of HLMC resulted from chemotaxis, rather than from chemokinesis (data not shown).

In addition, experiments with different concentration of ATF (aa 1–143) (10^−9^–10^−8^ M), which consists only of the uPAR-binding region of uPA and is devoid of enzymatic activity [18], demonstrated that this peptide retained its chemotactic properties (Fig. 3D). Taken together, these results indicated that uPA induced MCs migration by binding uPAR and stimulating its interaction with FPRs, most probably through the exposure of the uPAR_84–95_ epitope; indeed, the enzymatic activity of uPA was not primarily responsible for inducing HLMC chemotaxis.

## IV. DISCUSSION

A straight relation between cancer and flogosis has been extensively described, starting from the consideration that inflammatory cells are localized all around tumors [9, 34–36].

The interaction between cancer cells and their microenvironment are multiple and can result in both progression and arrest of tumor growth [37]. However tumor microenvironment is constituted not only by cells of the innate and adaptive immune system but also from stromal cells [38]. It has been described that stromal cells, from different tumors, are able to synthetize mRNA for diverse molecules, for example collagenase, matrix metalloproteases and proteases, such as plasmin, all responsible of the early stages of tumor growth through the degradation of the extracellular matrix components (ECM) that is one of the mechanisms that neoplastic cells use to invade the interstitial tissue [39].

Furthermore stromal cells, by producing chemoattractant molecules, recruit inflammatory cells into tumor sites, influencing them in a way that ultimately promotes cancer progression and prognosis [38,40,41]. In these context the detection of MCs within the tumor argues for their role in the modulation of neoplasia biology and hypothize a possible cross-talk with stromal cells localized within the tumor [27].

The three N-formyl receptors named FPR1, FPR2 and FPR3 are innate immune receptors belonging to the pattern recognition receptors and associated to G-proteins. Beside their involvement in inflammatory disorders FPRs have been described as regulating receptors involved in wound healing [42], angiogenesis [25], and myofibroblast activation occurring in the context of fibrotic disorders [24]. The involvement of FPRs in tumors has been studied by few authors and in some animal models where their role seems to differ in relation of the ligands or of the affected tissue [25]. We have recently showed that FPRs, as previously demonstrated in epithelial cells, could be involved in the pathogenesis of SSc, an autoimmune disease, through different mechanisms, including the interaction with the uPA-uPAR system [24]. In this paper we describe for the first time the expression of all the three FPRs in the human mast cell line HMC-1 (Fig. 1).

The uPA-uPAR system is an important and complex cellular recognition system that mediates different activities such as fibrinolysis, cell adhesion and migration, and tissue remodeling. In particular uPA, uPAR and the plasminogen activator inhibitor type-1 (PAI-1) are not only strongly upregulated in a wide variety of cancer types but their biological levels correlate with a poor neoplastic outcome and a more rapid tumor progression [22,43]. Nielsen and colleague described through in situ hybridization and immunohistochemical studies of invasive ductal breast cancer tissue that uPA, uPAR and PAI-1 are expressed in stromal cells immediately surrounding the invasive cancer cells [44].

We therefore studied the expression, at mRNA and protein levels, of uPA and uPAR in HMC-1 cells. HMC-1 cells, differently from basophils, synthetized uPA and expressed uPAR both in the intact and in a cleaved form (DII-DIII-uPAR), which is able to bind FPRs through the SRSRY sequence (residues 88–92) (Fig. 1). Interestingly, uPA itself can cleave uPAR in the DI-DII linker region, thus exposing the SRSRYsequence.

Moreover, by cytofluorimetric analysis using HLMC isolated from donors undergoing thoracotomy and lung resection, we confirmed that uPAR was expressed on the cell surface of the vast majority of HLMC (80–94%). We also found that enzymatically inactive uPA (DFP-uPA) and ATF, which is devoid of enzymatic activity, were potent chemoattractants for HLMC as well as the uPAR-derived peptide uPAR_84–95,_ containing the SRSRY sequence (Fig. 3). Thus, uPA released by MCs can act in an autocrine fashion both by cleaving uPAR and exposing the SRSRY sequence than, similarly to what happens in basophils, by triggering uPAR conformational changes able to mediate binding to FPRs and the transmission of signals from the cell surface to the inner domains involved in MCs chemotaxis.

We have previously demonstrated that the H. pylori-derived peptide Hp(2–20) stimulated eosinophil migration through the engagement of FPR2 and FPR3, and also induced production of VEGF-A and TGF-beta, two key mediators of tissue remodeling [32]. Thus, we evaluated the possibility that FPRs stimulation through uPA and uPAR_84–95_ triggering was able to induce synthesis and release of the Vascular endothelial growth factor-A (VEGF-A). VEGF-A is the most potent proangiogenic mediator known so far, involved in endothelial proliferation, migration, and survival. It also acts as a proinflammatory cytokine [45]. Both DFP-uPA and the uPAR_84–95_ peptide proved to be potent stimuli for VEGF-A synthesis and release. It has been demonstrated that, in murine models of glioma and meningioma, uPAR and cathepsin B knock-out inhibited angiogenesis by disrupting the JAK/STAT pathway-dependent VEGF expression [46,47]. Thus, uPAR could represent a useful target to inhibit VEGF-A mediated angiogenesis both in neoplastic and inflammatory diseases [48].

## V. CONCLUSION

Our work suggests the possibility that MCs, through the expression of the uPA/uPAR system and its interaction with FPRs, can be responsible of chronic inflammation, tumor progression and angiogenesis. Moreover, it can be hypothesized that stromal cells, by secreting chemotactic factors, can additionally contribute to the recruitment of MCs, for example, in the tumor surroundings.

Finally, the results described in this study may have practical implications in inflammatory and neoplastic disorders where MCs infiltration play a prominent role. In fact, several author recently suggested that the crosstalk between MCs and other tumor-infiltrating cells could be a potential target for anticancer therapies [13], or it is conceivable that agents acting on uPAR-mediated chemotaxis (i.e., by blocking the chemotactic epitope) may be used to modify the MCs driven inflammatory and tumor promoting reactions.
